# Glucocorticoid Receptor–Tethered Mineralocorticoid Receptors Increase Glucocorticoid-Induced Transcriptional Responses

**DOI:** 10.1210/en.2018-00819

**Published:** 2019-03-11

**Authors:** Caroline A Rivers, Mark F Rogers, Felicity E Stubbs, Becky L Conway-Campbell, Stafford L Lightman, John R Pooley

**Affiliations:** 1 Translational Health Sciences, Bristol Medical School, University of Bristol, Bristol, United Kingdom; 2 Department of Engineering Mathematics, University of Bristol, Bristol, United Kingdom

## Abstract

Mineralocorticoid and glucocorticoid receptors (MRs and GRs) constitute a functionally important dual receptor system detecting and transmitting circulating corticosteroid signals. High expression of MRs and GRs occurs in the same cells in the limbic system, the primary site of glucocorticoid action on cognition, behavior, and mood; however, modes of interaction between the receptors are poorly characterized. We used chromatin immunoprecipitation with nucleotide resolution using exonuclease digestion, unique barcode, and single ligation (ChIP-nexus) for high-resolution genome-wide characterization of MR and GR DNA binding profiles in neuroblastoma cells and demonstrate recruitment to highly similar DNA binding sites. Expressed MR or GR showed differential regulation of endogenous gene targets, including *Syt2* and *Ddc*, whereas coexpression produced augmented transcriptional responses even when MRs were unable to bind DNA (MR-XDBD). ChIP confirmed that MR-XDBD could be tethered to chromatin by GR. Our data demonstrate that MR can interact at individual genomic DNA sites in multiple modes and suggest a role for MR in increasing the transcriptional response to glucocorticoids.

Adrenal glucocorticoid hormones regulate a diverse range of physiological processes, including the stress response, immune function, and metabolic regulation, as well as adaptive behavioral and cognitive processes. Cortisol/corticosterone (CORT) are the predominant forms of glucocorticoid in humans and rodents, respectively. In addition to acute surges of CORT released during classical stress responses, circulating glucocorticoids fluctuate in basal nonstressed conditions during each 24-hour period. High CORT levels circulate during the active phase of the day, with significantly lower levels in the inactive phase establishing a characteristic circadian profile. Underlying this circadian profile is an ultradian profile with approximately hourly pulses of endogenous glucocorticoid detected in rat and human peripheral blood (1, 2), dialysates of subcutaneous extracellular fluid (3), and dialysates of extracellular fluid from discrete brain regions, including the hippocampus, of experimental rats (4).

The effects of CORT are mediated by two highly homologous receptors, the type I high-affinity mineralocorticoid receptor (MR) and the type II low-affinity glucocorticoid receptor (GR) (5). Glucocorticoid hormones are lipophilic and so can readily pass through the plasma membrane of target cells to bind to MRs and GRs, which act via genomic and rapid nongenomic mechanisms. Genomic effects are mediated by activated cytoplasmic receptors that translocate into the nucleus to precisely regulate patterns of gene transcription by integrating ligand activation with other signals, including DNA binding sequences, posttranslational modifications, and the presence of specific transcriptional regulatory factors (6). The biological complexity is further increased by the different affinities of the two receptors for the endogenous ligand CORT and the dynamic fluctuations of CORT levels. MR has near maximal occupancy with the very low CORT levels of the circadian nadir and remains maximally activated throughout the interpulse interval (7). In contrast, GR requires higher CORT levels associated with stress or the circadian peak (8) such that ultradian CORT pulses induce fluctuations in GR activity and DNA binding (9, 10), closely tracking the rise and fall of ligand concentration. Furthermore, MR and GR differ in body and brain expression patterns, with GR widely expressed throughout the brain and MR expression reportedly more restricted. Notably MR and GR are both highly coexpressed in limbic regions such as the hippocampus, amygdala, and some cortical areas (11, 12). Therefore, dynamic fluctuations in available ligand combined with differing receptor affinities and regional expression profiles provide a system with a complex array of potential outcomes.

GRs and MRs contain a 94% identical (13) highly conserved DNA binding domain that includes a proximal box (P-box) domain bearing three amino acid residues that directly contact the major groove of the DNA binding sequence and determine hormone response element specificity. GRs associate with specific genomic sites in multiple ways by directly binding to DNA as monomers or dimers or indirectly by tethering to specific genomic loci via interactions with other transcriptional regulatory factors such as AP-1 or nuclear factor *κ*B (6). MRs can recognize canonical palindromic GR binding sequences to which receptor dimers bind in head-to-head fashion. Specific binding sites have been identified for MRs and GRs in rat hippocampus (14), with overlapping hippocampal binding sites reported to range from 20% of total GR binding sites (475 out of 1925) (14) to 77% (10 out of 13) GR binding sites tested (15), although these studies cannot conclusively say that MR and GR binding occurs in the same cell types.

Therefore, we used chromatin immunoprecipitation with nucleotide resolution using exonuclease digestion, unique barcode, and single ligation (ChIP-nexus) methodology, chosen for its ability to achieve high-resolution ChIP sequencing (ChIP-seq) data (16) and discriminate close binding events, to compare MR and GR binding sites in the same cell type during a CORT pulse when both MR and GR are maximally activated. ChIP-nexus is one of several recently introduced improvements to ChIP-seq methodology that incorporate the use of *λ*-exonuclease digestion of captured DNA fragments 5′ to 3′ toward the cross-linked proteins to improve resolution of protein binding regions. On finding that GR and MR binding regions were highly similar, we demonstrated GR–MR tethering capable of increasing the dynamic range of GR-mediated transcriptional responses and revealing new insights into the modes of interaction between GRs and MRs.

## Materials and Methods

### Cell culture and reagents

Authenticated Neuro 2A (N2A) mouse neuroblast cells from the European Collection of Authenticated Cell Cultures [RRID: CVCL_0470 (17)] were purchased (January 2015) from Sigma-Aldrich (St. Louis, MO) and cultured up to passage 20, during which they showed consistent characteristic neuronal morphology and, upon differentiation stimulus (18), were capable of producing neurite projections (last tested September 2018). Cells were maintained in DMEM (Thermo Fisher Scientific, Waltham, MA) supplemented with 7.5% (v/v) fetal bovine serum (Gibco, Thermo Fisher Scientific), 0.3 mM cysteine-HCl, 0.4 mM l-alanine, 0.45 mM asparagine, 0.4 mM l-aspartic acid, 0.4 mM l-proline (Sigma-Aldrich), and 0.4 mM l-glutamate (Thermo Fisher Scientific) (18). After two washes with Dulbecco’s PBS (Thermo Fisher Scientific) cells were seeded in CSS media [DMEM with 7.5% (v/v) charcoal-stripped fetal bovine serum and the other supplements listed above] at 6 million per 15-cm plate for ChIP experiments and 0.25 million per well in six-well plates for RNA expression analysis. Cells were transfected ∼18 hours after seeding, treated with vehicle (ethanol, final concentration 0.01%) or 100 nM CORT (Sigma-Aldrich) 24 to 26 hours later, and, where appropriate, CORT was washed off by replacing the media twice with CSS-containing media.

### Plasmid constructs and transient transfections

ChIP-nexus experiments were performed with pC1-EGFP-rGR (gift from Gordon Hager, National Cancer Institute) (19) and pC1-mCherry-rMR (subcloned from pC1-EGFP-rMR, gift from David Pearce, University of California San Francisco). Plasmids encoded the EGFP fused to the N-terminal of rat GR cDNA and the mCherry protein fused to the N-terminal of rat MR cDNA inserted into pC1 vectors (Clontech/Takara Bio, Mountain View, CA). Five micrograms of pC1-EGFP-rGR and 5 µg of pC1-mCherry-rMR with 20 µL of jetPRIME^®^ (Polyplus Transfection, Illkirch-Graffenstaden, France) at a ratio of 1:2 (w/v) was used for transfection of cells in 15-cm plates.

For RNA expression analysis, untagged mouse GR cDNA in vector pC3 (gift from Gordon Hager, National Cancer Institute) and untagged mouse MR [gift of pcDNA4/TO-mMR from Diego Alvarez de la Rosa (20) in pSF-PGK-EMCV-Puro (Oxford Genetics, Oxford, UK)] were expressed. MISSION^®^ short hairpin RNA (shRNA) TRCN0000238462 (Sigma-Aldrich) targeting the 3′ untranslated region (UTR) of NR3C1 was coexpressed to knock down endogenous GR. Cells in six-well plates were transfected with 4 µL of jetPRIME^®^ [ratio of 1:2 (w/v)] per well and a total of 2 µg of DNA comprising 1 µg of shRNA vector plus 0.5 µg of GR and/or 0.5 µg of MR expression vectors, with pEGFP-C1 vector added as necessary to keep the total amount of transfected DNA constant.

For construction of the DNA binding domain mutations (XDBD), three amino acids in the P-box region (GR amino acids 446, 447, and 450 and MR amino acids 621, 621, and 625) were changed to tryptophans by overlapping PCR and traditional cloning methods (21). For GR, the DNA sequence GGAAGCTGTAAAGTC was changed to TGGTGGTGTAAATGG, and for the MR sequence GGCAGCTGCAAAGTC was changed to TGGTGGTGCAAATGG, which in both cases changed amino acids Gly-Ser-Cys-Lys-Val to Trp-Trp-Cys-Lys-Trp. For regular ChIP analysis the mCherry or EGFP was inserted upstream of the mouse MR-XDBD/GR-XDBD/wild-type cDNA sequences.

For construction of A640T MR mutants a single base of mouse MR (amino acid 640) was mutated to substitute alanine (GCT) with threonine (ACT) by overlapping PCR and traditional cloning methods. The encoded DNA binding domain D-loop amino acid sequence of these mutants was altered from EGQHNYLCAGRNDCIIDK to EGQHNYLCTGRNDCIIDK. DNA sequences of the constructs were confirmed by Sanger sequencing.

### RNA extraction and reverse transcription

Total RNA was extracted using an RNeasy Mini kit (Qiagen, Hilden, Germany) and treated with a TURBO DNA-free kit (Ambion/Thermo Fisher Scientific). One microgram of RNA was used for reverse transcription using an iScript™ cDNA synthesis kit according to the manufacturer’s instructions. Transcript levels were analyzed by real-time PCR using SYBR Green Fast PCR master mix (Applied Biosystems, Thermo Fisher Scientific) with *Mcm3ap* as an endogenous control that had constant expression with/without CORT treatment. Primers were designed with the PrimerQuest tool (Integrated DNA Technologies, Coralville, IA).

### ChIP-nexus

Hormone-treated EGFP-GR– and mCherry-MR–transfected N2A cells were fixed in 1% formaldehyde for 10 minutes and quenched with 3 mL of 1.25 M glycine for 5 minutes. Cells were washed three times with cold PBS, scraped off in PBS supplemented with cOmplete EDTA-free protease inhibitor cocktail (Roche, Basel, Switzerland), and centrifuged at 200*g* for 5 minutes at 4°C before freezing at −80°C. Chromatin was prepared from cell pellets by lysis in 1 mL of lysis buffer A1 (22) with protease inhibitors, douncing in a Wheaton Dounce homogenizer, and centrifugation at 3000*g* for 3 minutes at 4°C, with two further washes in A1. The final wash was in ELB buffer (23), and pellets were diluted 1:4 before sonication of 400-µL aliquots for four cycles (30 seconds on, 30 seconds off) in a Bioruptor plus (Diagenode, Liège, Belgium). After additions of 40 µL of Tris-buffered saline [0.5 M Tris (pH 8), 1.5 M NaCl], 40 µL of Triton X-100 10% (v/v), and 4.8 µL of 100 mM MgCl_2_, the aliquots were digested with 16 U of benzonase for 15 minutes at 25°C and the reaction was stopped with 80 µL of 0.5 M EDTA. After centrifugation for 15 minutes at 17,000*g*, 4°C, the supernatant from four aliquots was collected, diluted with buffer A2 (22), and preincubated with 100 µL of binding control magnetic agarose beads (ChromoTek, Planegg, Germany) for 2 hours at 4°C, before incubation with 100 µL of RFP-Trap^®^-MA [ChromoTek; catalog no. rtma-20; RRID: AB_2631363 (24)] or GFP-Trap^®^-MA [ChromoTek; catalog no. gtma-20; RRID: AB_2631406 (25)] overnight at 4°C with rotation. The ChIP combined with *λ*-exonuclease digestion (ChIP-exo) treatment exactly followed the Zeitlinger laboratory–published ChIP-nexus protocol (16). Samples were prepared with the NEBNext adaptor, NEBNext universal PCR primer, and NEBNext multiplex oligonucleotides for Illumina (indexes 1 to 9 of primer set 1) (21) and sequenced on an Illumina NextSeq processed using a 500 High Output Kit v2.

### ChIP

Chromatin was prepared as for ChIP-nexus up to the addition of 0.5 M EDTA and centrifugation. For each ChIP, 70 µg of chromatin was diluted 1:10 in ChIP dilution buffer (26), rotated 2 hours at 4°C with 25 µL of binding control magnetic agarose beads (Chromotek), and precleared lysate was rotated at 4°C overnight with 25 µL of RFP/GFP-magnetic agarose beads (Chromotek). Washing and elution of magnetic agarose bead complexes, reversal of crosslinks, digestion with ribonuclease (Roche) and proteinase K (Qiagen), and phenol-chloroform and chloroform (Sigma-Aldrich) extractions were as previously described (26). DNA was ethanol precipitated, washed in 70% ethanol, and resuspended in nuclease-free water prior to PCR amplification (21).

### Sequence analysis

Illumina ChIP-nexus sequencing data were quality filtered: reads were trimmed when the average quality of four bases was <20; reads <29 bp were dropped and reads were trimmed to 59 bp (9-bp barcode plus 50-bp sequence). Sequencing read alignment to the GRCm38/mm10 mouse reference genome was performed using Bowtie 2 short reads alignment programs [RRID: SCR_005476 (27)]. Random barcodes were registered and PCR duplicates containing the same random barcode were removed. Mapped data were filtered for mapping quality >20 to select uniquely aligned reads and minimize false-positives due to reads mapping to multiple genomic locations.

MACS2-enriched binding sites were called [RRID: SCR_013291 (28); false discovery rate <0.05, peak width of 100 bp], with background normalization data taken from input samples prepared from corresponding chromatin samples without immunoprecipitation. Binding sites were identified as within known genes, up to 5 kb upstream/downstream or intergenic when farther than 5 kb from a known gene. Peaks were considered to overlap when the genomic location of the MACS2 identified enriched binding sites directly overlapped by at least 1 bp.

Motif analysis for *de novo* motifs was performed with HOMER v4.7 [RRID: SCR_010881 (29)] with repeat masking. Genomic regions ±100-bp and ±200-bp MACS2 peak summits were analyzed for *de novo* motifs, and close matches were noted. Known motifs were also identified near the MACS2 peak summits using known motif probability matrices.

ChIP-nexus binding sites were predicted by the pipeline model-based analysis of ChIP-exo (MACE) v1.2 [RRID: SCR_005520 (30)]. To evaluate the relative positioning of GR and MR molecules genome-wide, we first identified all regions where there was binding for both molecules. For each of these overlapping sites, we used the midpoint of the GR site as a reference and tallied covered positions for both molecules relative to this point. Using this procedure, we obtained genome-wide coverage distributions for both GR and MR at overlapping sites.

### Statistical analysis

Reverse transcription (RT)/ChIP–quantitative PCR (qPCR) analyses were performed with a minimum of three independent biological replicates (n ≥ 3), and each quantitative PCR measurement was the mean of two separate qPCR values. RT-qPCR analyses were compared by one-way ANOVA with a Tukey multiple comparisons test, and data are represented as mean ± SEM. Statistical significance is designated as follows: *P < 0.05; **P < 0.01; ***P < 0.001; ****P < 0.0001.

## Results

### ChIP-nexus identifies GR and MR binding sites in N2A cells

**Figure 1.**
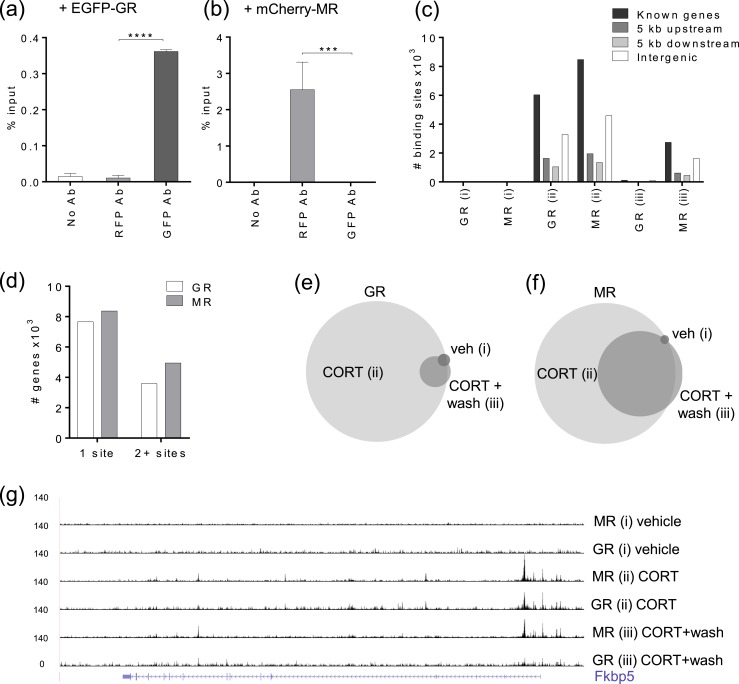
ChIP-nexus identifies GR and MR binding sites in N2A cells. (a and b) ChIP-qPCR showing specificity of (a) GFP and (b) RFP antibodies. N2A cells transiently transfected with EGFP-GR show the ChIP-qPCR signal detected by the GFP antibody with minimal cross-reactivity to the RFP antibody. Transfection with mCherry-MR shows the ChIP-qPCR signal detected by the RFP antibody with minimal cross-reactivity to the GFP antibody. EGFP-GR and mCherry-MR were cotransfected with shRNA to *NR3C1*-3′UTR to minimize possible endogenous GR effects, treated with 100 nM CORT for 20 min, and primers were located at the *Sgk1* gene. Data are represented as means relative to percentage input ± SEM (n = 3; one-way ANOVA with a Tukey test). (c) Graph shows the distribution of MACS2-identified GR and MR binding sites within known genes, 5 kb upstream, 5 kb downstream, and intergenic regions for each treatment group: (i) vehicle for 20 min, (ii) 100 nM CORT for 20 min, and (iii) 100 nM CORT for 20 min, washout, and further incubation for 40 min. (d) Graph shows the number of genes with single or multiple associated binding sites for GR/MR with treatment (ii). (e and f) Area-proportional Venn diagrams show the proportions of GR and MR MACS2 binding sites that directly overlap by at least 1 bp between treatments (i), (ii), and (iii). (g) University of California Santa Cruz Genome Browser image at the *Fkbp5* gene shows comparison of mapped MR and GR ChIP-nexus data for each treatment group. ***P < 0.001; ****P < 0.0001. Ab, antibody; veh, vehicle.

ChIP-nexus (16) was used to characterize genome-wide DNA binding sites for MR and GR in mouse neuroblastoma N2A cells. We chose to transiently transfect expression vectors for mCherry-MR and EGFP-GR because N2A cells have no MR expression, as is the case in many cell lines (31), whereas GR expression was present but showed minimal transactivation of a reporter gene (21). ChIP targeting the protein tags (32) mCherry and EGFP [Fig. 1(a) and 1(b)] achieved minimal cross-reactivity and excellent reproducibility, and it proved difficult to identify antibodies to the endogenous proteins suitable for ChIP when following the withdrawal of antibodies used in previous studies (14, 15). Preliminary testing of tagged EGFP-GR/mCherry-MR with an mouse mammary tumor virus promoter luciferase reporter construct (pFC31-luciferase) in N2A cells showed that the tagged GR and MR were functionally able to activate reporter gene transcription following CORT induction (21). The EGFP tag on GR and the mCherry tag on MR did not significantly alter transactivation potential in N2A cells, although subtle changes in response amplitude prompted the use of untagged MR and GR in all experiments when tags were not essential for ChIP. We assume that the tags do not alter DNA binding or interaction with other factors tethering the receptor to the DNA.

For the ChIP-nexus experiments, N2A cells were seeded in charcoal-stripped serum media before transient transfection with both mCherry-MR and EGFP-GR. After incubation for 24 to 26 hours, transfected cells were treated with (i) vehicle for 20 minutes, (ii) 100 nM CORT for 20 minutes, or (iii) 100 nM CORT for 20 minutes followed by a wash and media replacement without CORT for a further 40 minutes of incubation, with three replicates (n = 3) for each condition. Chromatin was prepared and treated according to the published ChIP-nexus protocol (16), with sequencing performed using lllumina NextSeq. Sequence data were quality filtered and mapped to mouse genome mm10. PCR duplicates containing the same random barcode at the same locus were removed, and mapped data were filtered on mapping quality (≥20) to retain reliably mapped reads and minimize mapping to highly repetitive regions, thereby reducing false-positives. For MR and GR ChIP-nexus, each treatment contained at least 8.9 million uniquely mapped reads (8.9 to 27 million), with individual replicates containing at least 1.9 million reads (1.9 to 12.54 million reads) (21). Sequence data are deposited in the National Center for Biotechnology Information’s Gene Expression Omnibus and are accessible through accession no. GSE115417 (33). Mapped reads were analyzed using MACS2 peak calling to identify enriched binding sites with a false discovery rate cutoff at 0.05 (34, 35).

The number of MACS2-enriched binding sites identified for MR and GR in each condition (replicates combined) and the number of known genes with associated binding sites (−5 to +5 kb) showed, as expected, that the greatest MR and GR binding was found after 20 minutes of CORT (treatment ii) (Table 1). Most of these binding sites were located within genes or within 5 kb upstream/downstream of genes. Only 28% of MR and 27% of GR binding sites were located in intergenic regions (>5 kb from a known gene) [Fig. 1(c)]. Many of the MR/GR-targeted genes were found to have more than one associated binding site (37% MR/32% GR) [Fig. 1(d)].

**Table 1.** MACS2 Enrichment Peaks Identified

Treatment	MACS2 Peaks		Known Genes (−5 kb to +5 kb) With Associated Peaks	
	MR	GR	MR	GR
(i) Vehicle	73	64	30	21
(ii) CORT	14,638	10,602	10,046	7312
(iii) CORT and wash	4885	246	3253	145

After CORT and washout (treatment iii), the binding of GR to chromatin was dramatically reduced to only 2.3% of the number of sites identified after CORT (treatment ii) [Fig. 1(e)], as expected, because a lower CORT concentration would reduce GR ligand binding and consequently GR occupancy. The appearance and disappearance of GR binding in vehicle, CORT, and washout treatments validates the hormone dependency of this dataset. A different binding pattern was seen for MR with 33% of the number of binding sites present even after CORT was washed out [Fig. 1(f)]. Retained MR binding is consistent with the higher affinity that MR has for glucocorticoids, prompting MR to associate more readily with DNA than does the lower-affinity GR. Most of the MR binding sites (77%) identified after washout (treatment iii) directly overlapped with MR binding sites found after CORT (treatment ii), consistent with retained MR binding at the same sites. Mapped ChIP-nexus data for GR and MR at the well-known GR-targeted gene *Fkbp5* is shown [Fig. 1(g)] and illustrates diminished GR binding after CORT washout (treatment iii), although substantial MR binding is retained after the same treatment. MACS2 analysis of the individual replicates treated with CORT (treatment ii) identified up to 6320 MR/7025 GR binding sites per replicate (21), with 5815 MR/1197 GR binding sites being reproducibly called in at least two out of three repeats with a direct genomic overlap of at least 1 bp.

**Figure 2.**
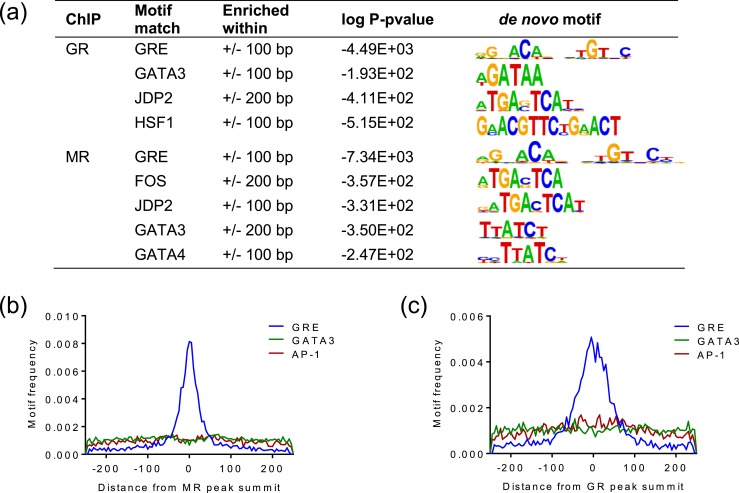
*De novo* and known transcription factor binding motifs near MR/GR binding sites. (a) *De novo* motifs identified in association with GR/MR binding sites within 100/200 bp of the MACS2 peak summits and corresponding close matches to known motifs. (b and c) Frequency of known motifs GRE, GATA3, and AP-1 around (b) MR and (c) GR MACS2 peak summits.

To discover motifs enriched at the MR/GR binding sites, MACS2 identified binding sites from combined replicates (MACS2 peak summits ±100 to 200-bp, treatment ii) were analyzed for *de novo* motifs using HOMER. The most significant motif was found to be 98%/97% (MR/GR sites) similar to the canonical glucocorticoid response element (GRE) and present in 58%/58% of sites. Other enriched motifs closely matched GATA transcription factors GATA3 (MR and GR) and GATA4 (MR), components of the AP-1 transcription factor FOS (MR) and JDP2 (MR and GR), as well as HSF1 (GR) [Fig. 2(a)]. The distribution of transcription factor binding sites using known motif matrices (21) around the MACS2 peak summits showed that only the GRE motif had a clear localization pattern that was centered, as expected, on the peak summit [Fig. 2(b) and 2(c)]. The enrichment of GATA3 motifs was found equally in binding sites containing GRE motifs (47% GR/50% MR) and sites without GRE motifs (51% GR/53% MR). Similarly, AP-1 motifs were enriched in both GRE-containing sites (11% GR/9.3% MR) and sites without GRE-like motifs (12.6% GR/11.5% MR). However, comparison of GR-only, MR-only, and GR+MR overlapping sites revealed slightly more enrichment of AP-1 and NF-1 known motifs in GR-only (18.5% AP-1/12.6% NF-1) and overlapping sites (19.2%/12.6%) compared with MR-only sites (16.3%/9.21%). Sites binding MR only were slightly more enriched for GATA3 and Phox2a known motifs (31.3% GATA3/10.3% Phox2a) than GR-only sites (28.1%/7.9%) or overlapping sites (28.7%/8.9%).

### MR and GR binding sites are highly similar

**Figure 3.**
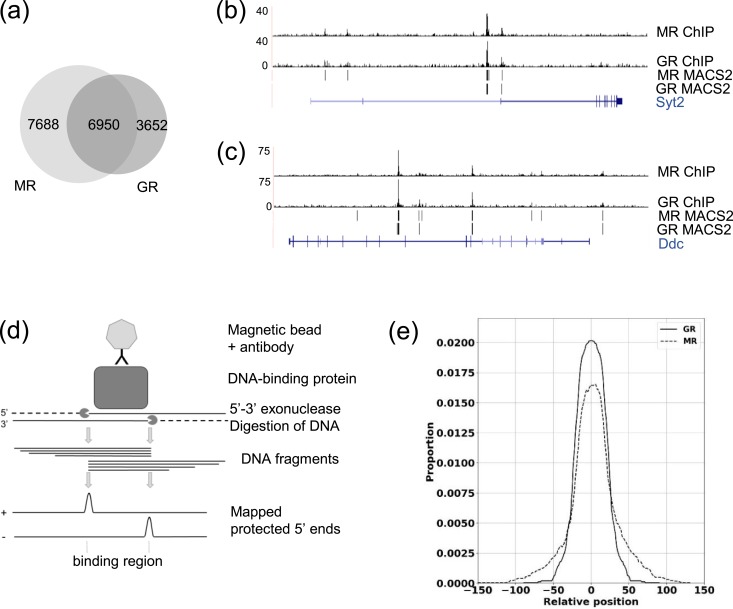
MR and GR binding sites are highly similar. (a) Area-proportional Venn diagram shows MACS2 binding sites for MR that directly overlap with GR binding sites by at least 1 bp after 20 min of CORT (treatment ii). (b and c) University of California Santa Cruz Genome Browser images of mapped ChIP-nexus data and MACS2 peaks for MR/GR in mCherry-MR– and EGFP-GR–transfected N2A cells after 20 min of CORT (treatment ii) at the (b) *Syt2* gene and (c) *Ddc* gene. (d) Diagram shows how *λ*-exonuclease digestion of ChIP fragments defines the binding regions. (e) Distribution of coverage for the MACE-predicted binding regions for GR and MR after CORT for 20 min (treatment ii) shows highly similar binding locations.

Comparison of MR and GR binding sites after CORT (treatment ii) showed a large intersection of binding sites to which MR and GR both bind, with 66% of GR binding sites having a direct overlap of at least 1 bp with MR binding sites [Fig. 3(a)–3(c)] (21). To clarify how the GR and MR binding sites relate to each other, a published ChIP-nexus data analysis pipeline, MACE (36), was used, which identifies the staggered ChIP-nexus borders characteristic of receptor binding regions by interrogating the plus and minus strand data separately. Identified border pairs flanking regions protected from *λ*-exonuclease digestion precisely define the DNA binding sites [Fig. 3(d)]. In total, 1184 MACE border pairs were identified with GR binding and 3285 with MR binding after CORT treatment. These highly confident binding sites correspond to 346 genes targeted by GR and 1225 genes by MR. The sizes of the regions identified were 10 to 60 bp for both MR and GR, with some larger regions (80 to 100 bp) for GR (21). To compare the predicted positions of GR and MR relative to each other on the chromatin, we evaluated the relative positioning of GR and MR molecules genome wide at overlapping binding sites. We obtained coverage distributions for both GR and MR relative to the midpoint of the GR binding region as a reference that, given the range of binding region sizes and the degree of overlap, covered positions up to 155 nucleotides away from the midpoint. The distributions were highly similar showing that GR and MR were recruited to precisely the same DNA regions [Fig. 3(e)].

### Differential effects of GR and MR on gene expression

**Figure 4.**
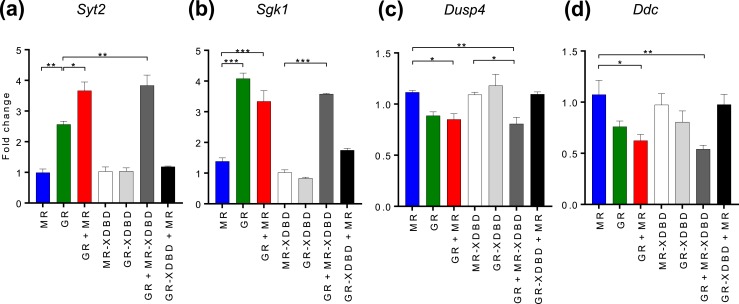
Differential effects of GR and MR on gene expression. (a–d) Graphs show (a) *Syt2* mRNA, (b) *Sgk1* mRNA, (c) *Dusp4* mRNA, and (d) *Ddc* nascent RNA changes in N2A cells with expressed wild-type MR and GR individually or combined, and MR-XDBD/GR-XDBD mutants individually or in combination with GR/MR. RT-qPCR data from N2A cells treated with 100 nM CORT for 2 h after transient transfection for 24 h with MR/GR/MR+GR expression plasmids and cotransfection of *NR3C1*-3′UTR shRNA to minimize endogenous GR effects. Data are represented as mean fold changes relative to EGFP-transfected controls ± SEM (n ≥ 3). *P < 0.05; **P < 0.01; ***P < 0.001.

Gene expression levels of several genes to which GR and MR both bound in N2A ChIP-nexus were measured by RT-qPCR. RNA was extracted from N2A cells treated with 100 nM CORT for 120 minutes and transiently transfected with expression constructs for untagged GR/MR alone, GR and MR together, or an EGFP control. shRNA targeting GR 3′UTR (*NR3C1*-3′UTR) was cotransfected in these experiments to minimize potential effects from endogenous GR. The inclusion of transfected sh*NR3C1*-3′UTR was shown to be effective at reducing endogenous GR mRNA (to 29%) and GR protein (to 33%) (21). We also measured reporter gene activation and transcriptional effects of endogenous GR in N2A cells, which were found to be minimal in N2A cells (21). Changes in gene expression levels included upregulation [*Syt2* and *Sgk1*, Fig. 4(a) and 4(b)] and downregulation [*Dusp4* and *Ddc*, Fig. 4(c) and 4(d)] relative to the EGFP-transfected control. When transfected alone, MR elicited only small changes in gene expression whereas GR caused greater gene induction/repression. Interestingly, when GR and MR were cotransfected the upregulation of *Syt2* mRNA was further increased compared with both MR and GR alone [Fig. 4(a)], and the downregulation of *Dusp4* mRNA and *Ddc* nascent RNA became significant compared with MR alone [Fig. 4(c) and 4(d)]. These augmented changes could possibly be due to additive effects of GR and MR individually, or they could represent interactions between GRs and MRs giving rise to altered activation/repression.

### MR DNA binding is not required for augmented effects

To investigate the mechanism mediating augmentation, MR and GR expression vectors were constructed with mutated DNA binding domains (XDBD) containing three amino acid substitutions in the DBD P-box, which is critical for DNA binding site recognition (37). This type of mutation has previously been demonstrated to prevent steroid receptor interactions with DNA (38), permitting us to interrogate whether augmentation required direct or indirect interaction with genomic binding sites.

Gene expression was measured using transfected wild-type and mutant GR and MR expression vectors alone and in combination. Augmented gene expression was observed for *Syt2* when mutant MR-XDBD was coexpressed with wild-type GR [Fig. 4(a)]. Similarly, the downregulation of *Dusp4* mRNA and *Ddc* nascent RNA, relative to MR expression, became significant when MR-XDBD was coexpressed with GR, as it was when wild-type MR was coexpressed with GR. Taken together, this demonstrates that MR did not require DNA binding to exert an effect. In contrast, when mutant GR-XDBD was coexpressed with MR, no significant changes in gene expression were observed, suggesting that GR binding is necessary for GR regulation. The finding that augmented gene expression could be induced even when MR was unable to bind the DNA demonstrated that augmentation was not due to the additive effects of MRs and GRs acting individually. Instead, the expression changes most likely result from interactions between GRs and MRs in a manner that is not dependent on MR binding DNA.

### MR is tethered to genomic DNA by GR

**Figure 5.**
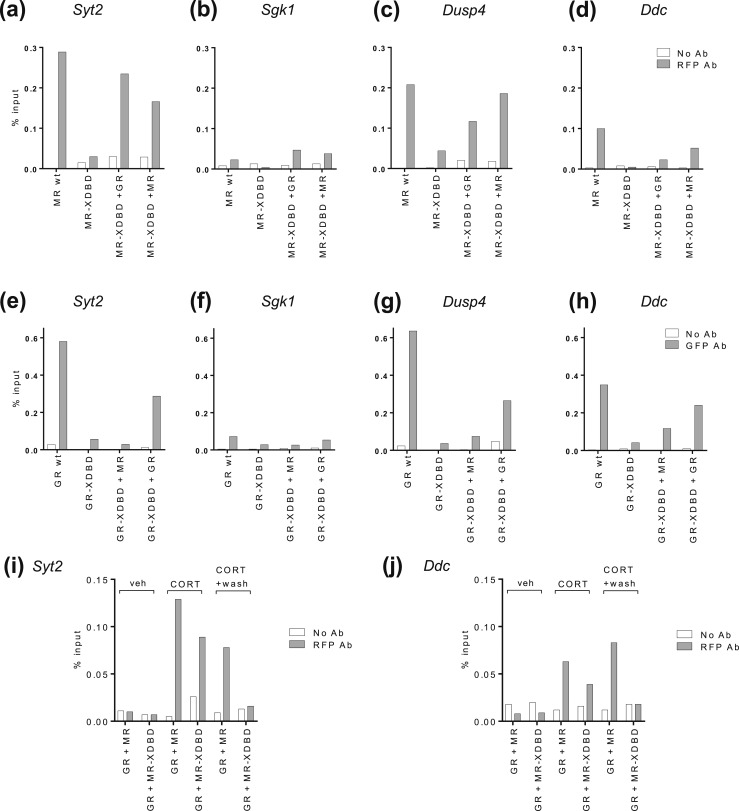
MR is tethered to genomic DNA by GR. (a–d) ChIP-qPCR shows mCherry-MR-XDBD binding to chromatin without/with cotransfected wild-type GR or MR at the (a) *Syt2*, (b) *Sgk1*, (c) *Dusp4*, and (d) *Ddc* binding sites. (e–h) ChIP-qPCR shows EGFP-GR-XDBD binding to chromatin with/without cotransfected wild-type MR or GR at the (e) *Syt2*, (f) *Sgk1*, (g) *Dusp4*, and (h) *Ddc* binding sites. N2A cells were treated with 100 nM CORT for 20 min (a–h). (i and j) ChIP-qPCR shows mCherry-MR-XDBD binding to chromatin at the (i) *Syt2* and (j) *Ddc* binding sites with treatments: vehicle for 20 min, 100 nM CORT for 20 min, or 100 nM CORT for 20 min, washout, and further incubation for 40 min. Data are taken from representative ChIP experiments (n = 3) expressed as a percentage of input. N2A cells were transiently transfected with mCherry-MR(wild-type)/mCherry-MR-XDBD/MR and EGFP-GR/EGFP-GR-XDBD/GR or an EGFP control, with shRNA to endogenous *NR3C1*-3′UTR. Ab, antibody; veh, vehicle; wt, wild-type.

To confirm that our XDBD mutations failed to bind N2A DNA and that the proposed MR–GR interaction was taking place on genomic DNA, further XDBD constructs were made with mCherry and EGFP tags suitable for ChIP testing. Wild-type mCherry-MR interacted with genomic DNA at the *Syt2*, *Sgk1*, *Dusp4*, and *Ddc* binding sites [Fig. 5(a)–5(d)], although apparently nonproductively (Fig. 4), and the binding at the *Sgk1* site was very weak, which may reflect a degree of epitope masking at this target. Mutant mCherry-MR-XDBD did not bind to the DNA on its own but was recruited to all four binding sites when coexpressed with wild-type GR [Fig. 5(a)–5(d)]. This shows that mutant MR can be tethered to genomic DNA by an interaction with GR, because it cannot bind to the DNA on its own.

Additional testing was performed with wild-type and mutant mCherry-MR and EGFP-GR in different coexpressed combinations. mCherry-MR-XDBD could be tethered to DNA binding sites by GR as well as by wild-type MR at the *Syt2*, *Dusp4*, and *Ddc* sites [Fig. 5(a), 5(c), and 5(d)]. EGFP-GR-XDBD could be tethered to DNA by wild-type GR at all sites [Fig. 5(e)–5(h)], but only by wild-type MR at the *Dusp4* and *Ddc* sites. As *Syt2* could load MR+MR-XDBD, GR+GR-XDBD, or GR+MR-XDBD and *Ddc* could load MR+MR-XDBD, GR+GR-XDBD, GR+MR-XDBD, and MR+GR-XDBD, we conclude that a single binding site appears capable of loading a variety of receptor complexes. The tethering of DNA binding–deficient forms of MR or GR by wild-type GR or MR has not previously been described. It is presently unclear as to the stoichiometry of the tethered complexes because GR and MR have typically been reported as dimers at DNA in crystal structures, but recent studies of GR DNA binding at a chromatinized DNA array in live cells found predominantly tetramers (19).

Because a CORT pulse followed by washout (treatment iii) diminishes GR binding [Fig. 1(c) and 1(e)], we predicted that if MR-XDBD was binding via GR, washout of CORT should diminish the binding of MR-XDBD observed when GR was also present. We performed further ChIP experiments using both GR and mCherry-MR(wild-type) or mCherry-MR-XDBD. Both MR and MR-XDBD appeared at the *Syt2* and *Ddc* binding sites after CORT induction but only MR(wild-type) binding was retained after CORT washout, confirming our prediction [Fig. 5(i) and 5(j)]. This further supports the idea that GR is tethering MR to DNA binding sites.

**Figure 6.**
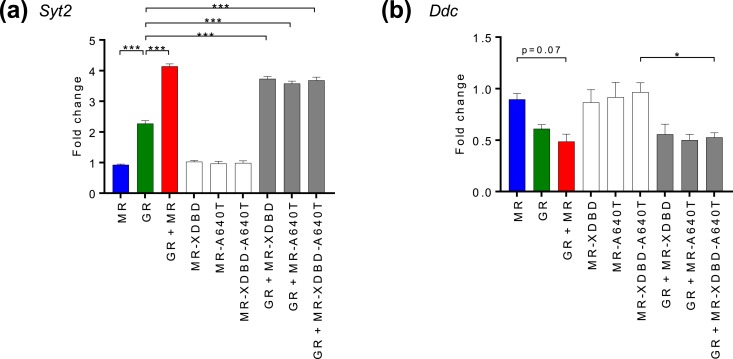
Effects of MR-A640T mutation on gene expression. (a and b) Graphs show (a) *Syt2* mRNA and (b) *Ddc* nascent RNA changes in N2A cells with expressed wild-type GR and MR/MR-XDBD/MR-A640T/MR-XDBD-A640T individually or combined. RT-qPCR data are shown from N2A cells treated with 100 nM CORT for 2 h after transient transfection for 24 h with MR/GR/MR+GR expression plasmids and cotransfection of *NR3C1*-3′UTR shRNA to minimize endogenous GR effects. Data are represented as mean fold changes relative to EGFP-transfected controls ± SEM (n ≥ 3). *P < 0.05; ***P < 0.001.

Tethering of MR-XDBD to GR could be via the classical dimerization interface, as a heterodimer, or by a different interface, and thus we created additional mutants to investigate how MR-XDBD and GR were interacting. Based on the rat GR A477T mutant with a disrupted D-loop dimerization interface, we made an equivalent Ala to Thr mutation (MR A640T) within the D-loop of MR that has a highly homologous DBD with identical sequence at this region. We observed augmented transcriptional response of the *Syt2* gene in the presence of both GR and MR even when MR was mutated in the D-loop dimerization interface (GR+MR-A640T/GR+MR-XDBD-A640T) (Fig. 6). A similar trend was observed at the *Ddc* gene. These results point to MR tethering by a novel form of MR–GR interaction, rather than heterodimerization.

Taken together, the gene expression and ChIP data findings show a new mode of interaction for MR, namely that MR can be tethered by GR to the DNA in such a way that it can function to alter gene expression levels. It has been known that MR can bind directly to palindromic GR binding sites, but these data show that it is also possible for MR to bind indirectly to the same DNA sites by GR tethering. Thus, multiple modes of MR interaction are possible on the same genomic sequences.

## Discussion

We propose a new mode of action for MR in which it can alter transcription when tethered to the DNA by GR. This MR–GR interaction is consistent with previous findings such as the presence of MR and GR *in vivo* on the same ChIP fragments (39) and earlier data showing inhibition of GR activation (40) or cooperative gene activation (41) with reporter constructs. The latter parallels our own observations of augmented gene activation/repression on two endogenous genes, although we now demonstrate augmentation within the native chromatin context, which is considerably more restrictive to receptor access than reporter plasmid DNA (42).

The mode of MR–GR interaction appears to be via a novel form of MR–GR interaction, rather than heterodimerization, because MR A640T D-loop mutants were equally able to direct augmented gene expression changes. The equivalent amino acid to A640 has been described as a central mediator in the interaction of GR homodimers, and this residue supports the identical interface between MR and GR in the classical heterodimer model of interaction (40, 43). MR–GR interactions via interfaces other than the classical dimerization interface have been reported, including interaction between the N-terminal domain of MR with GR (44) and between MR and the ligand binding domain of GR, which was necessary for MR-mediated nuclear translocation of a translocation-deficient GR variant (45).

Importantly, we show that GR–MR interactions do occur in endogenous genomic contexts and that they have functional consequences on gene transcription, including increasing gene upregulation and downregulation. In this way, the tethered MR–GR interactions we identified can enhance the magnitude of the transcriptional response to glucocorticoids.

We show in neuroblastoma cells that CORT-activated MR is recruited to binding sites that frequently harbor GRE motifs (58%). This is in contrast to a study in kidney cells (46) where only 7.4% of MR binding sites were found to contain GRE-like motifs, leading to the suggestion that MR was likely to interact with chromatin via other transcription factors instead of directly binding to DNA sites. As different cell types and ligands (aldosterone vs CORT) were used in the two studies, the discrepancy may be due to cell type–specific variations in the chromatin landscape, as previously described (47–49), and/or ligand-specific differences in MR binding. It is also possible that overexpression of the MR protein may have resulted in increased MR binding at GRE-containing sites in our study. However, consistent with our findings for a high proportion of MR binding sites containing GRE motifs, an MR ChIP-seq study in rat hippocampus reported a high proportion of GRE motifs underlying MR binding loci (14).

GR DNA binding has been shown to be a highly dynamic process (50) with rapid exchange of GR at binding sites. *In vivo* GRs are ligand activated only during ultradian peaks of glucocorticoid secretion (7) and during the stress response. Therefore, genomic GR–MR interactions can only occur at these times, and we expect GR–MR augmented regulations to show pulsatile profiles in a similar way to GR alone (51). Within such a dynamic responsive system, enhanced transcriptional responses through GR–MR interactions provide increased sensitivity to CORT signaling as well as an increased magnitude of response. However, we do not yet know how widespread MR–GR tethering is, as we have only assessed this at selected sites and measured the outcome for expression of a few candidate glucocorticoid target genes.

MR and GR are strongly coexpressed in the hippocampus, and decreased MR levels or MR/GR ratios are implicated in stress and psychiatric diseases (52, 53). The tethering of MR to GR provides a new insight into how the signaling via these receptors could be restricted when MR levels diminish. It is now recognized that MR and GR coexpress in adipocytes, bone osteoblasts, monocytes and macrophages, and in cells of the kidney collecting duct (54). Although our present work was performed in a neuronal cell line, similar functional MR–GR interactions may additionally occur in other cell types. Access of glucocorticoids to MR is controlled by the balance of two related enzymes, 11*β*-hydroxysteroid dehydrogenase (11*β*-HSD) 1 and 11*β*-HSD2 (55). Glucocorticoid action through MR is physiologically normal for some cell types such as osteoblasts (56) and hippocampal neurons (57) where 11*β*-HSD1 is dominant and active glucocorticoids are locally regenerated from inactive metabolites. However, 11*β*-HSD1 dominance may also arise as a consequence of disease state (54) in cell types where 11*β*-HSD2 activity would normally dominate and ensure MR selectivity to aldosterone by destruction of active glucocorticoids. Thus, our finding of augmented GR-mediated responses as a consequence of MR tethering may be mechanistically relevant beyond the brain.

Specifically, we observed changes in the regulation of *Syt2*, a synaptotagmin Ca^2+^ sensor responsible for exocytosis, shown to be required for ensuring fast feed-forward inhibition at *γ*-aminobutyric acid–ergic junctions, at least in the cerebellum (58). Our results identify *Syt2* as a novel glucocorticoid-responsive gene and show MR–GR augmentation achieves increased expression of *Syt2*, and consequently tethered MR may be necessary for ensuring efficient inhibitory tone in some neural microcircuits.

We present a novel mode of action for MR that increases the magnitude and sensitivity of GR-modulated responses, at least for *Ddc* and *Syt2*. Further investigation is warranted to assess the genome-wide impact of tethered MR on gene expression changes, as this mechanism could prove to be highly important in understanding hypothalamic–pituitary–adrenal activity and regulation.

## Abbreviations:

11*β*-HSD: 11*β*-hydroxysteroid dehydrogenase
ChIP: chromatin immunoprecipitation
ChIP-exo: chromatin immunoprecipitation combined with *λ*-exonuclease digestion
ChIP-nexus: chromatin immunoprecipitation with nucleotide resolution using exonuclease digestion, unique barcode, and single ligation
ChIP-seq: chromatin immunoprecipitation sequencing
CORT: cortisol/corticosterone
GR: glucocorticoid receptor
GRE: glucocorticoid response element
MACE: model-based analysis of chromatin immunoprecipitation–exo
MR: mineralocorticoid receptor
N2A: Neuro 2A
P-box: proximal box
qPCR: quantitative PCR
RT: reverse transcription
shRNA: short hairpin RNA
UTR: untranslated region
XDBD: DNA-binding domain mutant
